# Pleural effusion metastatic papillary thyroid carcinoma with dedifferentiation: a case report and review of the literature

**DOI:** 10.3389/fonc.2025.1575821

**Published:** 2025-05-28

**Authors:** Jialing Xu, Bin Huang, Huajuan Ruan

**Affiliations:** ^1^ Department of Medical Oncology, The First People’s Hospital of Xiaoshan District, Hangzhou, Zhejiang, China; ^2^ Department of Pathology, The First People’s Hospital of Xiaoshan District, Hangzhou, Zhejiang, China; ^3^ Department of Pathology, The First People’s Hospital of Lin’an District, Hangzhou, Zhejiang, China

**Keywords:** pleural effusion, metastasis, papillary thyroid carcinoma, dedifferentiation, clinicopathology

## Abstract

Pleural effusion metastatic papillary thyroid carcinoma with dedifferentiation is a rare clinical presentation. We present the case of a 78-year-old female patient who developed chest tightness, shortness of breath, and cough without an obvious cause over the past two weeks. Chest computed tomography (CT) revealed consolidation of the right lung with massive pleural effusion. Cytological examination of pleural effusion revealed malignant tumor cells. Cell wax block analysis showed that the tumor was a nested, papillary, and single-cell structure, and epithelioid cell atypia was obvious. Nuclear grooves or intranuclear pseudoinclusion bodies were not observed. Immunohistochemical results were negative for thyroglobulin (TG) and thyroid transcription factor 1 (TTF-1), partially positive for paired box gene 8 antigen(PAX-8), and a Ki-67 proliferation index of 30%. Further immunohistochemical tests were positive for Braf-V600E and Ber-EP4, and the thyroglobulin serologic test was 265.90 µg/mL. The patient had undergone cervical lymph node dissection for right papillary thyroid cancer 21 years ago, developed lung metastases 4 years ago, and received targeted therapy. This case reminds us that metastatic pleural effusion tumors with atypical pathological morphology and immunohistochemical expression should be comprehensively evaluated in combination with clinical, immunohistochemical, and serological tests, which may help in the diagnosis and treatment of clinicians and pathologists.

## 1 Introduction

Papillary thyroid carcinoma(PTC) is the most common thyroid malignancy and is usually characterized by slow growth and a good prognosis (1). Pleural effusion metastatic papillary thyroid carcinoma is a relatively rare clinical manifestation (2) and is often associated with the progression and metastasis of thyroid cancer. However, when the tumor metastasizes, especially with the formation of pleural effusion, the patient’s prognosis may deteriorate significantly (3). The clinical symptoms of pleural effusion include dyspnea, cough, and chest pain. Cytological examination of pleural effusion showed that not all of them had papillary structures, nuclear sulcus, or pseudonuclear inclusion bodies, and not all of them had positive TG and TTF-1 expression on immunohistochemistry (4, 5). At present, there are few reports of systemic distant metastatic papillary thyroid carcinoma with dedifferentiation (6–8). Here, we report a case of pleural effusion metastatic papillary thyroid carcinoma with dedifferentiation(PEMPTCD), which provides a reference for clinical and pathological studies and avoids errors in diagnosis and treatment.

## 2 Case report

This patient was a 78-year-old female. Two weeks prior, the patient had no obvious cause of chest tightness, shortness of breath, cough, white phlegm, or hoarseness, and no discomfort such as hemoptysis, chills, fever, nausea, vomiting, chest pain, headache, or dizziness. The patient was hospitalized in the Binjiang Maternal and Child Health Hospital (Zhejiang, China), but her symptoms remained unabated, and she was transferred to our hospital (Zhejiang, China) for treatment. At admission, the patient was conscious, with a poor mental state, normal sleep, normal bowel movements, and no significant weight gain or loss. Physical examination revealed surgical traces of thyroid cancer on the right side of the neck, with no palpable tumor, no palpable lymph nodes in the neck and other superficial regions, and a weak right breath sound on lung auscultation.On January 5, 2025, a low-dose computed tomography (CT) scan of the lung revealed right lung consolidation with a large pleural effusion. The results of tumor serology were as follows: Alpha-fetoprotein <2.00 ng/mL (normal range 0.89-8.78 ng/mL), carcinoembryonic antigen 2.72 ng/mL (normal range 0.00-5.00 ng/mL), glycoantigen 19-9>1200.00 U/mL (normal range 0.00-37.00 U/mL), Glycoantigen 125 is 236.50 U/mL (normal range 0.00-35.00 U/mL).Previous history: The patient underwent surgery for a right thyroid tumor in 2003 and was pathologically diagnosed with papillary thyroid carcinoma with cervical lymph node metastasis (7/42). On December 29, 2020, owing to the discovery of multiple nodules in both lungs on CT examination, puncture examination of the right lung tumor was performed at Run Run Shaw Hospital (Zhejiang, China). Pathological examination revealed lung metastasis through a typical PTC, with immunohistochemical expression of TG, TTF-1, and PAX-8.

**Figure 1:**
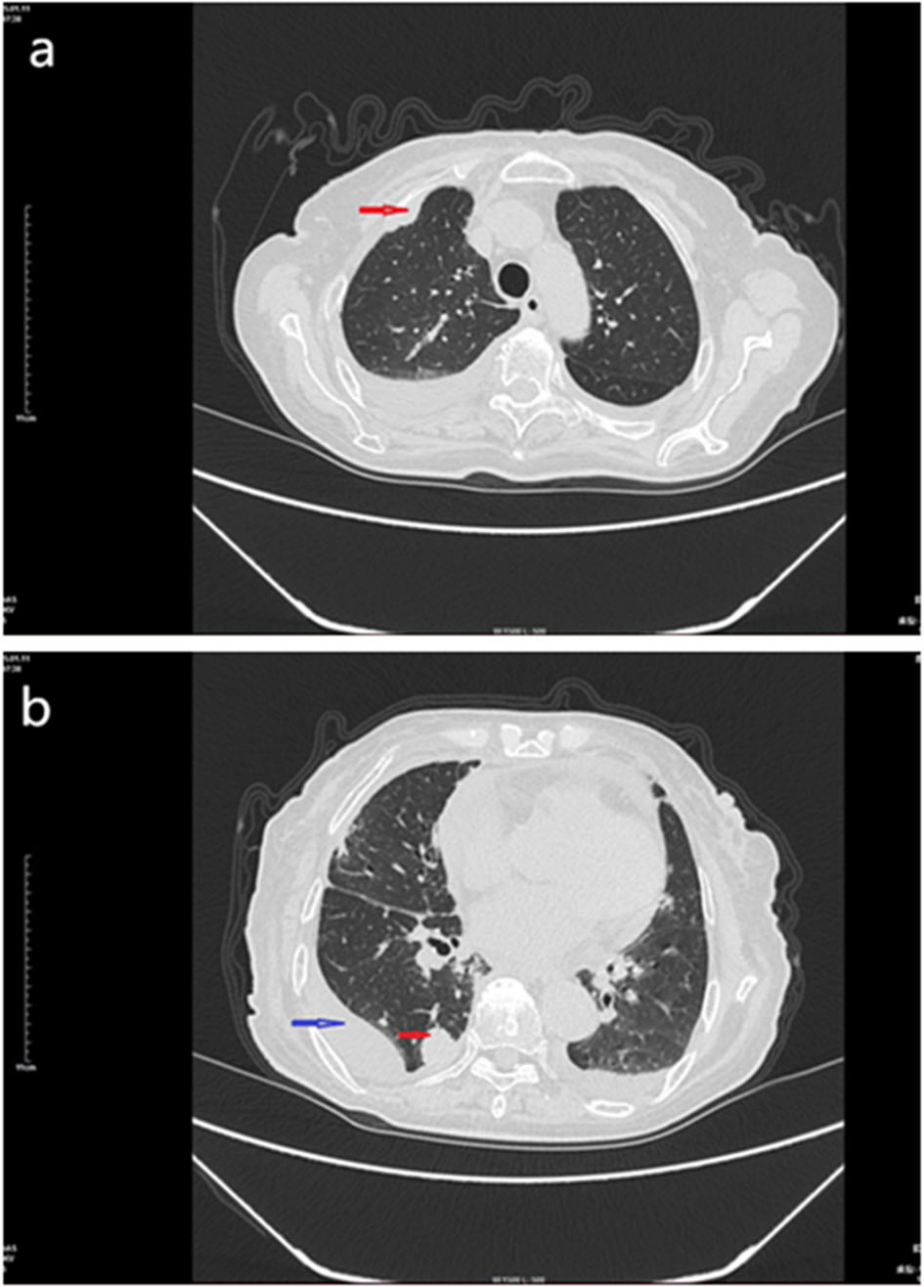
CT scan of the chest mass, January 11, 2015. **(a)** Enhanced chest CT showing a soft tissue mass in the right lung pleura (red arrow). **(b)** Enhanced chest CT showing a mass in the right lung (red arrow) and pleural effusion (blue arrow).

On January 11, 2025, enhanced chest CT (**Figures 1a, b**) revealed a mass in the lower lobe of the right lung, involving the right pulmonary hilum and right pleura, and multiple small nodules in both lungs with a small amount of pleural effusion. Mass in the left hilar lung. No significant tumors were found in other parts of the body.

**Figure 2:**
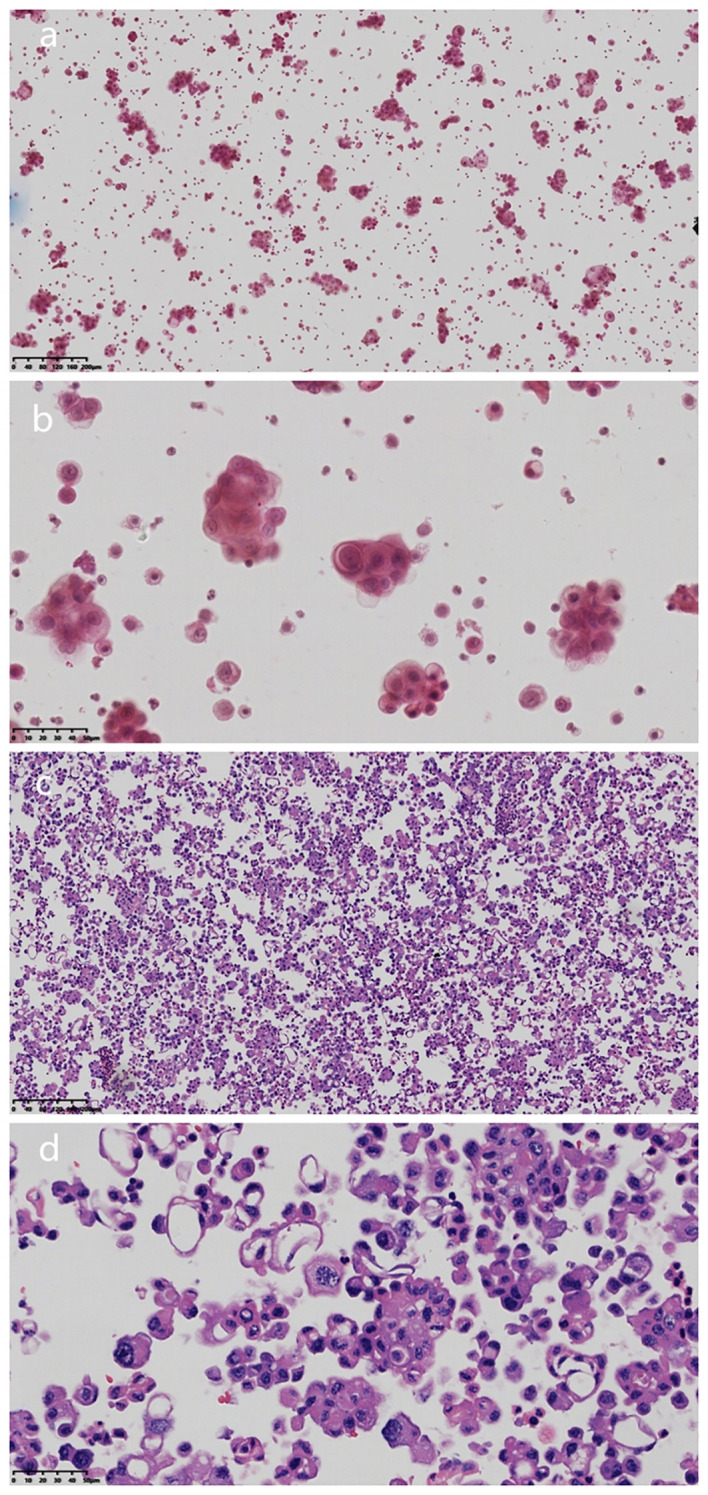
HE characteristics of pleural effusion cytology and cell wax block examinationa. **(a)** Microscopically, the atypical cells in the smear showed single or nested structure (Magnification, x100; The scale bar, including 200µm; H&E staining). **(b)** Tumor cells were different in size, with large nuclei, high ratio of nuclei to plasma, and small nucleolus staining (Magnification, x100; The scale bar, including 200µm; H&E staining). **(c)** HE sections of cell wax block showed that the tumor was nested, papillary and single cell (Magnification, x400; The scale bar, including 50µm; H&E staining). **(d)** Epitheloid cells had obvious atypia, high nucleo-plasma ratio, and coarse chromatin (magnification, x400; scale bar, 50 µm).

Puncture and drainage of the right pleural effusion were performed on January 5, 2025, and the pleural effusion was sent to the pathology department for cytological examination. Under the microscope, the atypical cells in the smear showed single or nest-like structures with different cell sizes, large nuclei, a high nucleo-plasma ratio, and small nucleoli (**Figures 2a, b**). Cell wax blocks and immunohistochemical examinations were simultaneously performed. The production process of the cell wax block mainly includes the following steps: Use a fine needle to extract an appropriate amount of body fluid (50 ml, concentrate the cells by the centrifuge method, centrifuge speed 2000 revolutions per minute, 3–5 minutes. The centrifuged cell mass was fixed with 10% neutral formalin for 8 h and then transferred to an embedded box for dewatering and waxing. Wax-impregnated cells were placed in an embedding box and embedded in melted paraffin. The paraffin blocks were cured and cut into 3µm thin slices.Sections were subjected to routine pathological HE staining and Envision immunohistochemical staining for further pathological analysis and diagnosis.

**Figure 3:**
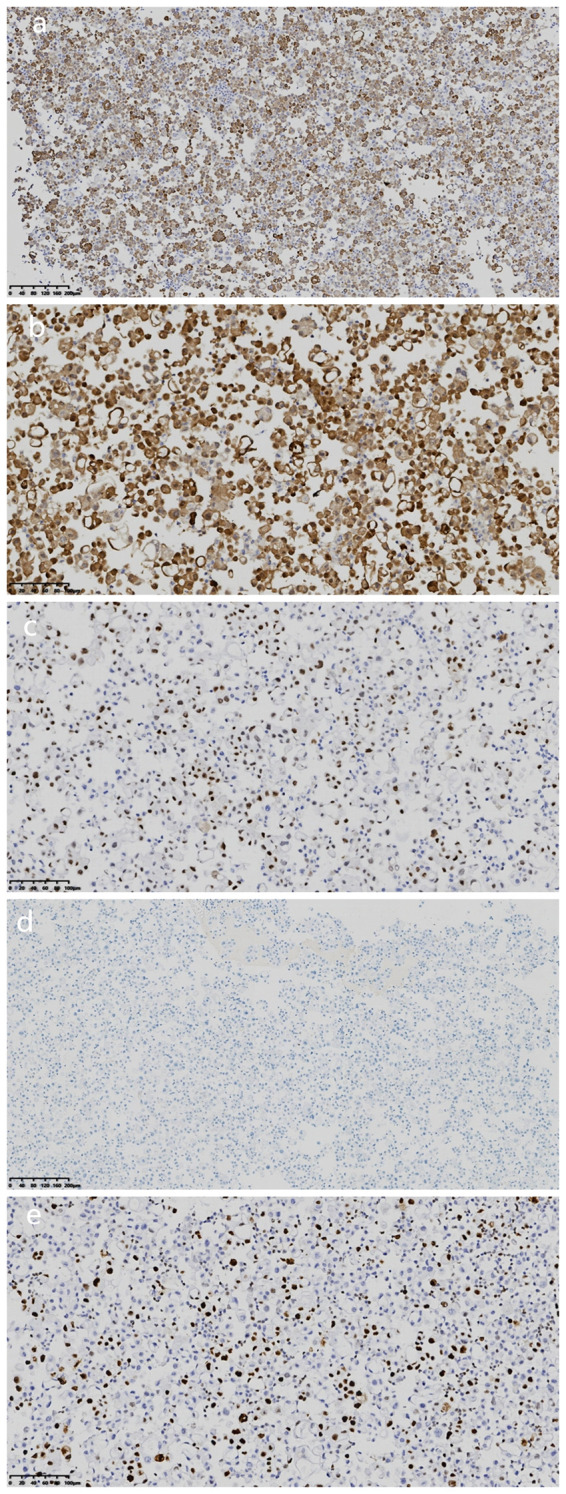
Immunohistochemical analysis of cell wax block. **(a)** Immunohistochemical staining revealed that the tumor cells were positive for CK5/6. (magnification, IHC x100; Envision). **(b)** Immunohistochemical staining revealed that the tumor cells were positive for calretinin. (magnification, IHCx200; Envision). **(c)** Immunohistochemical staining revealed that the tumor cells were partial positive for Pax-8 (magnification, IHC x200; Envision). **(d)** Immunohistochemical staining revealed that the tumor cells were negative for TG (magnification, IHCx100; Envision). **(e)** Ki-67 proliferation index was 30%(magnification, IHCx200; Envision).

**Figure 4:**
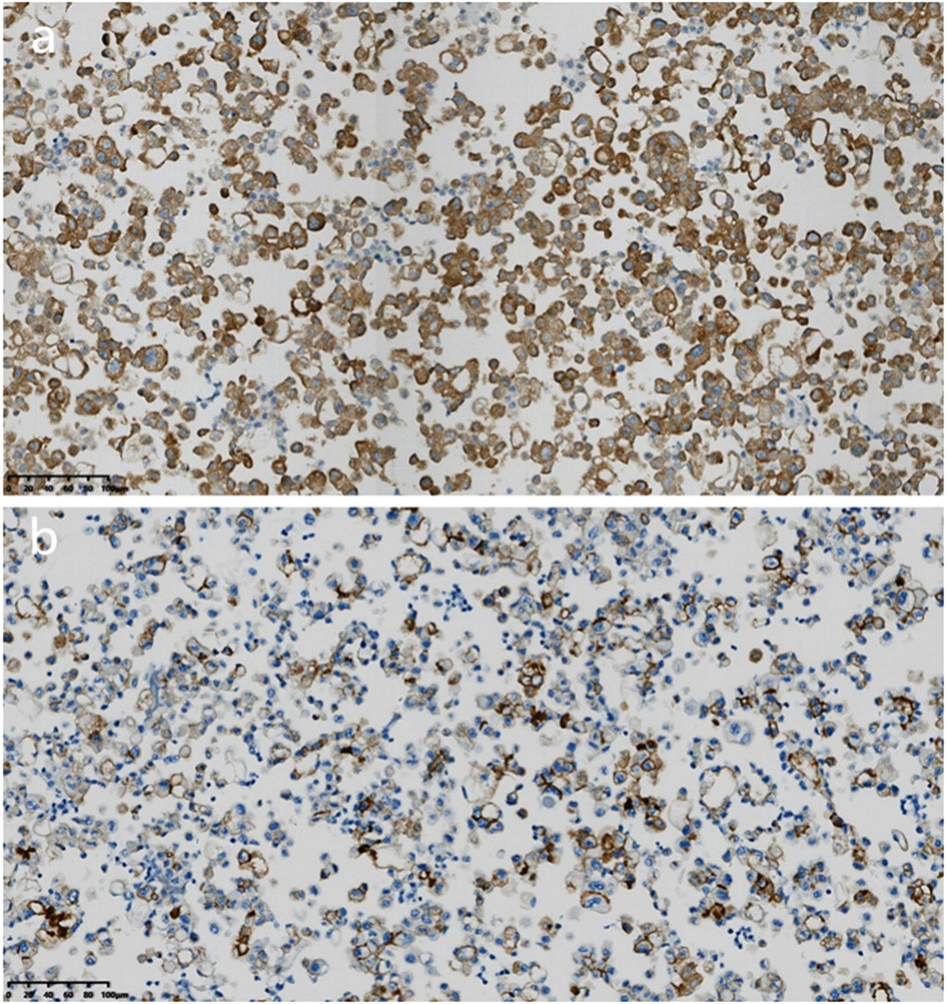
Immunohistochemical analysis of cell wax block. **(a)** Immunohistochemical staining revealed that the tumor cells were positive for Braf-V600E (magnification, IHCx200; Envision). **(b)** Immunohistochemical staining revealed that the tumor cells were positive for Ber-EP4 (magnification, IHCx200; Envision).

Microscopic examination of the cell wax block HE section showed that the tumor was a nested, papillary, and single-cell structure (**Figure 2c**). Epithelioid cells showed obvious atypia, large size, round or oval shape, high nucleo-plasma ratio, coarse chromatin (**Figure 2d**), and no nuclear grooves or intranuclear pseudoinclusion bodies. Envision immunohistochemical analysis showed that CK5/6 (**Figure 3a**), calretinin (**Figure 3b**), CK7, CA125 were positive, and PAX-8 (**Figure 3c**) and HBME1 were partly positive. TG (**Figure 3d**), TTF-1, p40, Napsin A, WT-1, D2-40, PR, ER, CK20, CDX-2, Villin, and CEA were negative, and the Ki-67 proliferation index was 30% (**Figure 3e**). Additional Braf-V600E positive (**Figure 4a**) and Ber-EP4 was positive (**Figure 4b**). Serologic retesting of thyroglobulin was 265.90 µg/mL (normal range 3.50-77.00 µg/L, patients usually around 20 µg/mL). The pathological diagnosis was pleural effusion metastatic papillary thyroid carcinoma with dedifferentiation.

The patient received three times of oral ^131^I therapy (60,100,150 mg, respectively) after PTC operation in 2003. After the diagnosis of lung metastasis in December 2020, she was started on dabrafenib capsules (50–100 mg, bid) due to a Braf-V600E mutation, along with two additional doses of oral ^131^I (200 mg each, The patient herself requested a higher dose, which was not recommended at the time). After the patient took a total of 400mg of ^131^I, the whole body scan(WBS)showed no iodine uptake, and the tumor did not shrink, so the ^131^I treatment was stopped.Anlotinib capsule (8 mg once daily) was added in 2023, but the patient did not take it regularly.After the diagnosis of PEMPTC, the patient is currently receiving dabrafenib capsules (50mg,twice daily) and anlotinib capsules (8mg,once daily). After 3months of follow-up, the patient died of respiratory failure due to the unresolvable tumor and increasing pleural effusion.

## 3 Discussion

Thyroid cancers include papillary carcinoma, follicular carcinoma, medullary carcinoma, anaplastic carcinoma, and others. PTC is the most common thyroid malignancy and usually shows slow growth and good prognosis (1). Pleural effusion metastatic papillary thyroid carcinoma (PEMPTC) is a relatively rare clinical manifestation (2) at an advanced clinical stage. Simultaneously, the prognosis of patients may deteriorate significantly (3). At present, cases of systemic distant metastatic papillary thyroid carcinoma with dedifferentiation are relatively rare (6–8), and systematic studies are lacking to discuss its clinical features, diagnostic criteria, and treatment plans. We report a case of pleural effusion metastatic papillary thyroid carcinoma with dedifferentiation, and review the literature to improve the diagnosis and treatment of this disease by clinicians and pathologists.

Metastatic papillary thyroid carcinoma with dedifferentiation in the submaxillary region, liver, breast, pleura, and small intestine is often accompanied by complex clinical manifestations (6–8). Pleural effusion presents with dyspnea, cough, and chest pain, similar to lung cancer or chronic obstructive pulmonary disease, making early diagnosis and accurate treatment challenging. At the same time, when dedifferentiated, the pathologic HE morphology of propapillary thyroid carcinoma is different, showing fusiform, pleomorphic, giant cell morphology, etc. (6–8), which makes diagnosis more difficult.

Papillary thyroid carcinoma usually shows the expression of typical immunohistochemical markers such as TG, TTF-1 and Pax8, etc. However, in metastatic thyroid cancer with pleural effusion, the absence of thyroid markers can occur (4, 5), and the expression of NapsinA can also occur in pericardial effusion (9), which confounds the diagnosis. Hosoda et al. (8) summarized 14 cases, among which five cases showed that TG and TTF-1 were negative, and five cases showed positive results for Pax8. Immunohistochemical results of the dedifferentiated tumor cells in this case showed that TG and TTF-1 were negative, suggesting that conventional immunohistochemical results should not be relied upon for diagnosis of thyroid cancer metastasis. This atypicity not only increases the risk of misdiagnosis, but may also lead to unnecessary treatment. Although Pax8 positivity strongly suggests thyroid origin, gynecological and nephrogenic tumors need to be ruled out (10).; further immunohistochemistry is needed to confirm this. The supplementary immunohistochemical analysis results showed the presence of the Braf V600E mutation and the positive expression of Ber-EP4, combined with the patient’s thyroglobulin serological examination was 265.90 µg/mL(About 20µg/mL in normal steady state), further supporting the diagnosis of metastatic papillary thyroid carcinoma (11). At the same time, positive expressions of CK5/6 and Calretinin was found in this case, which needs to be differentiated from pleural mesothelioma (12, 13). The immunohistochemical expression of Pax-8, BrafV600E, and Ber-EP4 can be differentiated from mesothelioma.

Previous study believe that there is a correlation between ^131^I treatment and anaplastic transformation of thyroid cancer (8), the reason is that DNA damage of tumor patients is caused by ^131^I treatment, and the combination of p53 mutation and DNA damage induced by ^131^I treatment will lead to anaplastic transformation. Among the 14 patients with anaplastic transformation at distant metastatic sites summarized by Hosoda et al. (8), 11 received ^131^I treatment. The patient in this case also received ^131^ I treatment five times, and it is considered that the dedifferentiation of tumor cells after metastasis may be related to ^131^I treatment.

When PTC progresses to MPTCD, it indicates that the disease has entered a more aggressive stage and the prognosis is significantly worse (2, 3). Targeted drugs and immunotherapy have been greatly applied in anti-tumor therapy (14, 15): if there is a BRAF V600E mutation, BRAF V600 mutant kinase inhibitors can be used to inhibit the BRAF V600 mutant kinase, block the BRAF/MEK/ERK signaling pathway, and inhibit tumor cell proliferation and survival. Multi-targeted tyrosine kinase inhibitors antiangiogenic drugs, which act by inhibiting angiogenesis and tumor cell proliferation, are mainly used in the treatment of advanced malignant tumors. If PD-L1 is positive, monoclonal antibodies or combination therapy can be used. This suggests that evaluation of the molecular profile of the tumor could lead to more individualized treatment options for patients in the setting of distant metastasis and dedifferentiation. In this case, the tumor showed BRAF mutation at the time of lung metastasis, and ^131^ I was not effective. The patient survived for 4 years with dabrafenib and anlotinib combination therapy, indicating that targeted drugs have a certain effect in the treatment of advanced thyroid cancer (16). Current pleural effusion metastatic cancer cells showing BRAF mutation continue to be treated with dabrafenb and anlotinib; unfortunately, we did not perform PDL1 testing. After 3 months of follow-up, the patient died of respiratory failure due to the unresolvable tumor and increasing pleural effusion.

In conclusion, through this case, we realized that in the clinical and pathological diagnosis of complex and atypical tumor manifestations, it is necessary to combine clinical, pathological, and immunohistochemical evaluations to optimize the diagnosis and treatment of patients. When the tumor has distant metastasis and the original treatment is ineffective, we can speculate that PTC has transformed into PTCD, and confirm it by pathology. Since there are few cases of MPTCD, more clinical data need to be accumulated for an in-depth study. In addition, this case provides insight for future research and a valuable basis for clinicopathological diagnosis and treatment.

## Data Availability

The raw data supporting the conclusions of this article will be made available by the authors, without undue reservation.
